# Painless Leg Weakness Revealing a Falcine Meningioma in the Postpartum Period

**DOI:** 10.7759/cureus.116782

**Published:** 2026-09-23

**Authors:** Brent A Spiteri, John Capodilupo, David Spiteri

**Affiliations:** 1 Physical Medicine and Rehabilitation, Kansas City University, Kansas City, USA; 2 Biomedical Sciences, Grand Valley State University, Allendale, USA; 3 Physical Medicine and Rehabilitation, Corewell Health William Beaumont University Hospital, Royal Oak, USA

**Keywords:** falcine meningioma, intracranial mass, lumbar radiculopathy mimic, meningioma craniotomy, neurologic localization, painless leg weakness, parasagittal meningioma, postpartum low back pain, upper motor neuron

## Abstract

Low back pain is among the leading causes of disability worldwide and is common in pregnancy, which can lead a clinician to a musculoskeletal diagnosis. We describe a 41-year-old postpartum woman who presented to an outpatient office with mechanical low back pain and painless weakness of the left lower extremity that had caused three falls. A thorough history established that the weakness was non-radicular and unaccompanied by sensory loss, and the neurologic examination localized the deficit to the upper motor neuron rather than to a lumbar root or peripheral nerve. Magnetic resonance imaging of the brain revealed a large extra-axial falcine mass consistent with a meningioma, exerting a mass effect on both frontal lobes, greater on the right, which accounted for the contralateral left foot weakness. The patient underwent urgent right frontal craniotomy with resection, and her weakness resolved completely. This case emphasizes that a disciplined history and physical examination, grounded in neuroanatomy, can distinguish upper from lower motor neuron pathology and can uncover a life-threatening intracranial lesion in a patient whose presentation initially suggests a benign postpartum musculoskeletal cause.

## Introduction

Low back pain is one of the leading causes of disability in most countries, as shown by a systematic analysis comparing the prevalence of 310 diseases and injuries from 1990 to 2015 [1]. Low back pain during pregnancy is likewise common, with a reported prevalence of up to 68% in the United States [2]. Mechanical low back pain in pregnancy is largely attributable to maternal weight gain, increasing lumbar lordosis, and ligamentous laxity within the pelvis driven by hormonal changes such as relaxin. Because this presentation is so common, low back pain and lower extremity symptoms in the peripartum period are readily attributed to a lumbar or pelvic source, which can anchor the clinician and delay recognition of a more serious process.

Painless weakness broadens the differential well beyond the lumbar spine. It may arise from disorders of the neuromuscular junction such as myasthenia gravis or Lambert-Eaton syndrome, from intrinsic muscle disease such as myopathy, or from upper motor neuron disorders of the central nervous system, including multiple sclerosis, stroke, brain tumors, and amyotrophic lateral sclerosis. Less commonly, it reflects a lower motor neuron lesion at the root or peripheral nerve. Distinguishing among these possibilities depends on a working knowledge of upper and lower motor neuron anatomy. A lower motor neuron forms the efferent limb of the peripheral nervous system; in the lower extremity its cell bodies lie in the anterior horn of the spinal cord, and its axons exit as ventral roots to innervate skeletal muscle. An upper motor neuron has its cell body in the motor cortex and synapses in the brainstem or spinal cord onto the lower motor neuron [3,4]. The spinal cord terminates near the first lumbar vertebra, below which mixed nerve roots containing motor, sensory, and pain fibers form the cauda equina. Because these roots are mixed, compression of a lumbar root typically produces pain and sensory loss in the corresponding dermatome in addition to myotomal weakness. Weakness without any accompanying pain or sensory change therefore points away from a lower motor neuron lesion and toward the upper motor neuron.

## Case presentation

History

A postpartum 41-year-old woman presented to an outpatient office with low back pain and weakness of the left lower extremity. She had delivered approximately one month earlier without complications. During the third trimester, she had experienced mechanical, localized low back pain that worsened with transitional movement and pointed toward the sacroiliac joint region; this pain improved over the weeks following delivery, leaving only mild residual discomfort at the time of evaluation. Around the same time, she began to notice left lower extremity weakness, which resulted in three falls after her delivery. She denied any injury or head trauma from the falls. She also denied visual complaints, headaches, dysphagia, dizziness, lightheadedness, and bowel or bladder incontinence and reported no upper extremity symptoms. On further questioning, she denied any radicular pain down the left lower extremity despite the weakness. One week earlier, another physician had ordered a lumbar spine MRI and discussed a possible epidural steroid injection for a presumptive diagnosis of lumbar radiculopathy; however, she again denied leg pain and noted that her back symptoms had improved since that visit.

Her family history was negative for neurofibromatosis type 2, multiple endocrine neoplasia type 1, and familial meningiomatosis. The patient had never smoked and did not use smokeless tobacco, alcohol, or drugs. She was independent with basic and instrumental activities of daily living.

Physical examination

Neurologic examination revealed normal cranial nerves with no nystagmus. Strength was intact in all four extremities except for isolated weakness of the left tibialis anterior and extensor hallucis longus (dorsiflexors of the left foot) and the left iliopsoas (hip flexor), each graded 4/5. Strength was preserved in the gluteal, hamstring, adductor, quadriceps, and gastrocnemius groups. Gait was normal in pattern, though slower in cadence despite the weakness noted on manual muscle testing. Sensory examination was normal throughout, including the thoracic spine. Reflexes were brisk and symmetric in all four extremities. Plantar responses were equivocal with no Babinski sign, and there was no clonus. Finger-to-nose and heel-to-shin testing were normal, with no dysmetria or ataxia in the upper or lower extremities. Lhermitte, Hoffmann, and palmomental signs were negative. Straight and reverse straight leg raises were normal. Musculoskeletal examination revealed minimal discomfort over the left sacroiliac joint, with no pain on percussion, flexion, or extension of the lumbar spine, no tenderness over the sciatic notch, and full, painless range of motion of both hips. The calves were soft and non-tender, with a negative Homans sign bilaterally.

Investigations and diagnostic reasoning

The physical examination narrowed the differential generated by the history. Screening maneuvers for upper motor neuron pathology, including the Babinski, Hoffmann, palmomental, and Lhermitte signs, along with clonus, dysmetria, and ataxia testing, were all normal in this patient. Deep tendon reflexes help distinguish a central from a peripheral lesion: the brain exerts a net inhibitory influence on the cord, so a hemispheric lesion may produce contralateral hyperreflexia; a spinal cord lesion may produce hyperreflexia with a Babinski sign and clonus; diffuse areflexia suggests Guillain-Barré syndrome or peripheral neuropathy; and isolated asymmetric reflex loss suggests radiculopathy, plexopathy, or mononeuropathy. In this patient, the reflexes were brisk, which is not uncommon in the peripartum state, but they were symmetric and unaccompanied by pathologic signs.

Manual strength testing was interpreted using the Medical Research Council scale, which grades muscle strength from 0 to 5 against applied resistance [5]. The left dorsiflexors (tibialis anterior, extensor hallucis longus, and extensor digitorum longus) and the iliopsoas were each graded 4/5. The dorsiflexors are innervated by the deep fibular nerve, a branch of the common fibular division of the sciatic nerve, with root contributions primarily from L4 and L5 [6]. The iliopsoas musculotendinous unit (IPMU) receives multisegmental innervation, reflecting the combined structure of the psoas major, psoas minor, and iliacus muscles [7]. The psoas muscles are supplied by direct branches from the anterior rami of L1-L3, and the femoral nerve (L2-L4) supplies the iliacus [7]. Because the sensory and motor roots converge at the intervertebral foramen to form these nerves, the distal portion carries both motor and sensory. The absence of radicular pain or sensory disturbances made lumbar radiculopathy and peripheral nerve involvement less likely in this patient.

Combining the history of painless, non-radicular weakness with an examination showing weakness in an L4 to L5 distribution but a preserved patellar reflex, normal sensation in all dermatomes, negative straight and reverse straight leg raise, and brisk symmetric reflexes raised the possibility of an upper motor neuron lesion. Multiple sclerosis, which can worsen in the postpartum period, was considered [8], as were central tumors and stroke. An MRI of the brain and cervical spine with and without contrast was ordered; the patient was already scheduled the same evening for a non-contrast lumbar spine MRI ordered by another physician.

Imaging findings

MRI of the brain with and without gadolinium demonstrated a large extra-axial mass measuring approximately 52.1 by 63.6 by 46.9 mm, arising from the falx cerebri near the vertex (Figures 1, 2). The mass was predominantly high in T2 signal, becoming heterogeneous where it contacted the falx, with areas of low T2 and intermediate T1 signal, and it occupied the right side of the falx primarily, with a small extension across the midline (Figure 2). Regional mass effect was most notable on the superior aspects of both frontal lobes, greater on the right, accounting for the left foot weakness, with minimal subcortical edema along the margins. There was no hydrocephalus and no acute or subacute infarct. The cervical spine MRI showed no findings that correlated with the history or examination.

**Figure 1:**
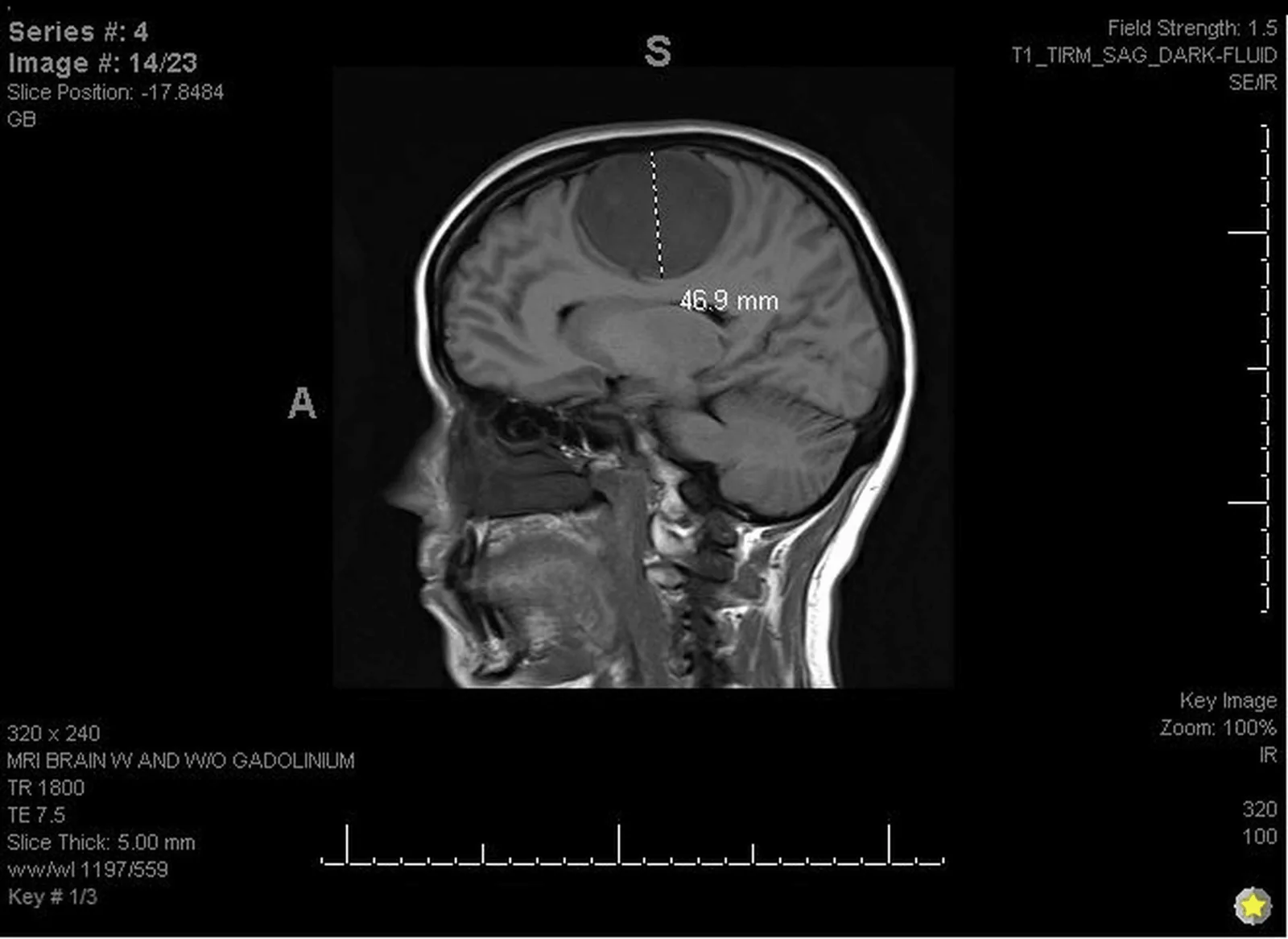
MRI of the brain demonstrating a large extra-axial falcine mass near the vertex measuring 46.9 mm in depth. Sagittal view of an MRI of the brain. A denotes anterior, and S denotes the superior aspect of the patient.

**Figure 2:**
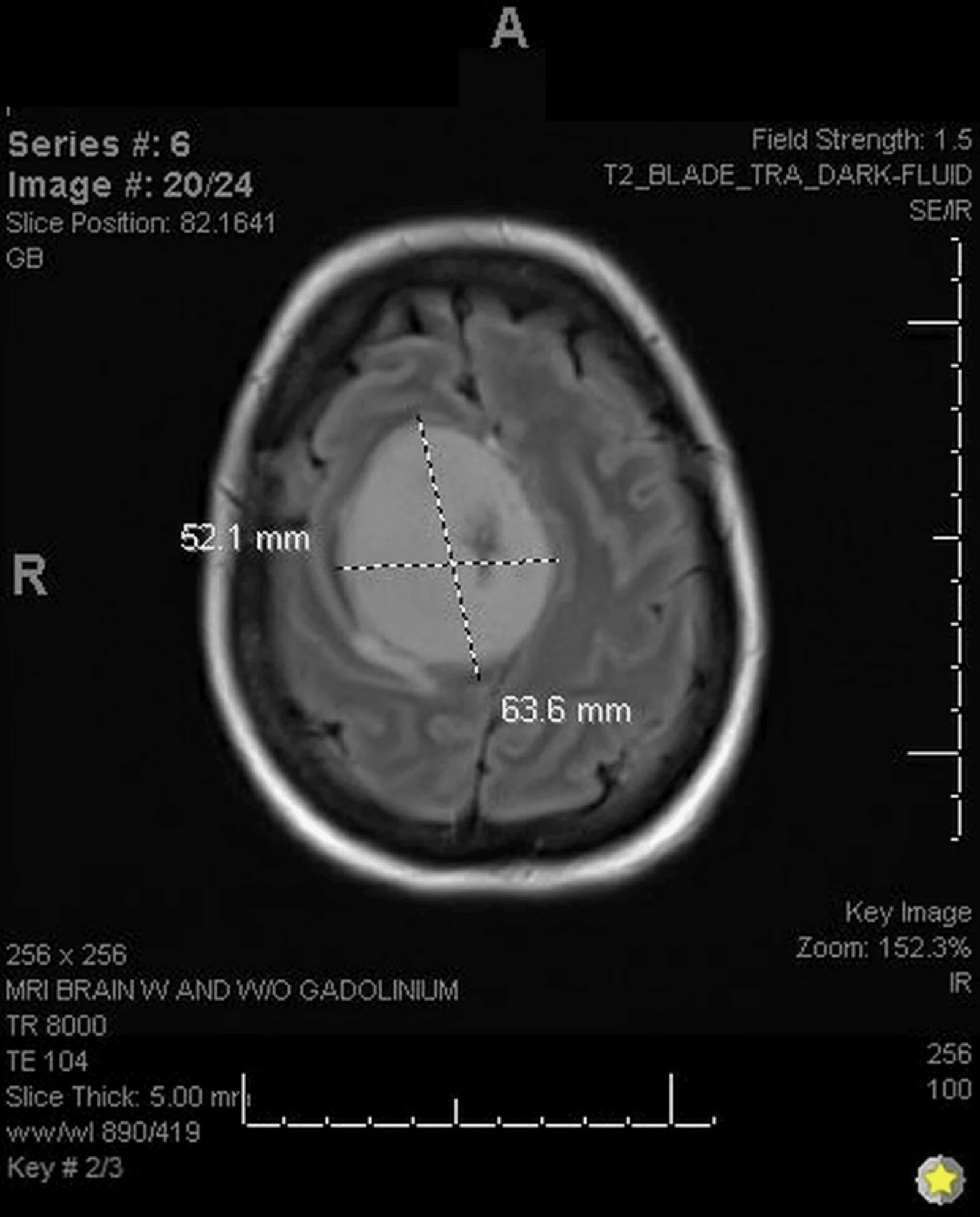
T2-weighted MRI showing the extra-axial mass measuring approximately 52.1 mm by 63.6 mm. Axial view of MRI of the brain. R denotes the patient’s right side. A denotes the anterior or front side of the patient.

MRI visualizes the brain, spinal cord, and vascular anatomy in three anatomical planes, and gadolinium contrast improves delineation of inflammation, tumors, infarcts, and vascular abnormalities [9]. Signal characteristics reflect proton relaxation properties: high T2 signal can indicate water, edema, fat, blood, or most tumors, whereas high T1 signal can indicate fat, melanin, blood, proteinaceous fluid, or chelated gadolinium [10]. The lesion location and signal characteristics were consistent with meningioma, and post-contrast imaging showed homogeneous enhancement, a pattern seen in roughly 85% of meningiomas (Figure 3) [11]. Contrast-enhanced imaging further excluded demyelinating diseases such as multiple sclerosis and cerebrovascular disease, and the accompanying cervical spine MRI was included to raise sensitivity for multiple sclerosis under the McDonald criteria by evaluating for cervical cord lesions [12]; none were found.

**Figure 3:**
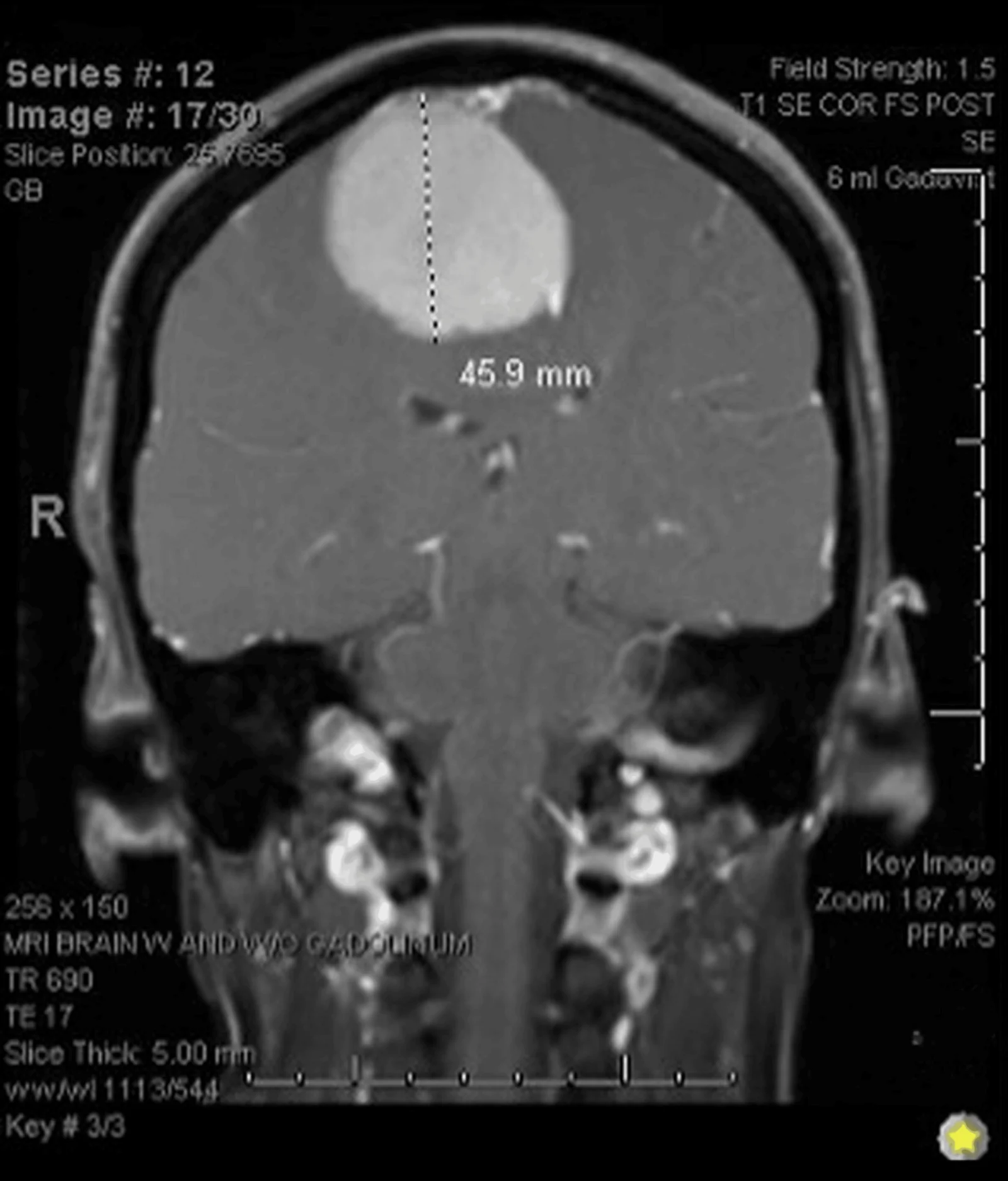
T1-weighted post-contrast MRI showing homogeneous enhancement of the tumor. Coronal view of MRI of the brain. R denotes the right side of the patient.

Treatment and outcome

A head CT was obtained to determine whether the tumor was confined to the meninges or had invaded the skull; it confirmed that the mass was contained within the meninges without bony involvement. A cerebral angiogram was performed to map the arterial supply and to assess the need for preoperative embolization. The head CT and cerebral angiogram were performed by the neurosurgeon at an outside institution for mapping of the tumor prior to surgery. The patient then underwent a right frontal craniotomy with resection of the tumor, with careful protection of the arteries, sinuses, and adjacent brain. At postoperative re-evaluation, she reported improved strength and denied radicular symptoms, visual complaints, or gait instability. On examination, the previous left lower extremity weakness had resolved to 5/5 in all muscle groups, and the remainder of the neurologic examination was normal.

## Discussion

This case illustrates how a structured history and examination, interpreted through neuroanatomy, can redirect a diagnosis from a common musculoskeletal cause to a life-threatening intracranial lesion. Two features of the history were decisive. First, the low back pain followed the expected trajectory of peripartum sacroiliac pain, improving after delivery, which made an active lumbar or pelvic source of the weakness unlikely. Second, and more important, the weakness was painless and non-radicular throughout pregnancy and the postpartum period. Because lumbar roots are mixed nerves, a compressive radiculopathy severe enough to cause a visible motor deficit would ordinarily produce dermatomal pain or sensory loss; their absence argued strongly against a lower motor neuron process.

The examination reinforced this reasoning. Weakness confined to one limb in both a proximal (iliopsoas, L2 to L4) and distal (dorsiflexors, L4 to L5) distribution, combined with a preserved patellar reflex indicating intact L4 root function, was difficult to reconcile with a single radiculopathy. Normal sensation in all dermatomes and negative provocative maneuvers further reduced the likelihood of nerve root irritation, while the asymmetry of a single affected limb made a diffuse myopathy improbable. Brisk reflexes, although partly expected in the peripartum state, were consistent with an upper motor neuron contribution. Taken together, these findings moved central pathology to the front of the differential and justified brain imaging rather than the lumbar injection that had been contemplated.

Meningiomas are common extra-axial tumors that grow slowly and can reach a large size before producing focal deficits, particularly when they arise parasagittal or from the falx and compress the medial motor cortex serving the contralateral leg [11]. In this patient, the right-sided falcine mass compressed the superior frontal lobes, greater on the right, producing contralateral left foot and hip flexor weakness that mimicked an L4 to L5 process. The postpartum context added a plausible but misleading alternative, since multiple sclerosis can worsen after delivery [8]; contrast-enhanced brain and cervical spine imaging excluded demyelinating and cerebrovascular disease and secured the diagnosis. The complete resolution of weakness after resection supports the causal link between the tumor and the deficit and underscores the value of timely recognition.

Limitations

The histopathology was not available to the authors following tumor resection. Consequently, the WHO grade of the meningioma could not be determined, nor could its histological features be characterized.

## Conclusions

A postpartum patient with mechanical low back pain and painless left leg weakness was found to have a large falcine meningioma requiring urgent craniotomy, after which her weakness resolved. The key to the diagnosis was not advanced technology but a disciplined history and physical examination interpreted through an understanding of upper and lower motor neuron anatomy, which distinguished a central lesion from the far more common lumbar or pelvic causes suggested by the presentation. Clinicians should be alert to painless, non-radicular weakness as a signal to look above the spine, since early recognition of such a lesion can prevent progression to cerebral herniation and death.
